# Highlights on the risk of pulmonary tuberculosis in patients on ibrutinib treatment: Case report and literature review

**DOI:** 10.1002/jha2.80

**Published:** 2020-09-19

**Authors:** Solo Traoré, Mehdi Roumila, Pirayeh Eftekhari, Hassan Farhat, Fatiha Merabet, Oumar Guira, Philippe Rousselot, Reza Azarian, Caroline Besson

**Affiliations:** ^1^ Training and Research Unit in Health Sciences Joseph Ki ZERBO University Ouagadougou Burkina Faso; ^2^ Department of Internal Medicine Yalgado Ouedraogo Teaching Hospital Ouagadougou Burkina Faso; ^3^ Department of Hematology‐Oncology Versailles Hospital Center Le Chesnay France; ^4^ Department of Pneumology Versailles Hospital Center Le Chesnay France; ^5^ Faculty of Medicine University Paris‐Saclay UVSQ Montigny‐le‐Bretonneux France; ^6^ French Regional Pharmacovigilance Center Fernand‐Widal Hospital‐APHP Paris France; ^7^ CESP, INSERM U1018 University Paris‐Saclay UVSQ Villejuif France

The management of low‐grade lymphoid malignancies has been profoundly modified by the use of ibrutinib, a Bruton tyrosine‐kinase inhibitor (BTKi). However, BTKi can induce the occurrence of serious infections [1]. Here, we present the case of a patient who developed pulmonary tuberculosis (TB).

An 85‐year‐old man from Ivory Coast (West Africa), who had lived in France since the age of 36, but frequently traveled to Africa, had been followed for a marginal‐zone lymphoma (MZL) associated with an immunoglobulin M component and a translocation (11;18) since 1997. He had a medical history of hypertension and Guillain‐Barre syndrome and no history of HIV. He received chloraminophene between 1997 and 2001 due to anemia and medullary involvement, six cycles of rituximab‐cyclophosphamide in 2008 after the recurrence of MZL localized to the intestine, and six cycles of rituximab‐bendamustine in 2011 for a gastric relapse. In 2018, following the recurrence of a large mesenteric MZL, he was treated with 420 mg ibrutinib orally per day combined with monthly rituximab treatment (375 mg/m^2^) [2]. A computerized tomography (CT) scan performed after 6 months of therapy showed a reduction of the tumour diameter from 9 to 3.5 cm contrasting with the appearance of pulmonary micronodules and mediastino‐hilar adenomegalies (Figure 1). The patient complained of cough and evening fever. Bacteriological analysis of the bronchial aspirate revealed the presence of *Mycobacterium tuberculosis* complex in culture, confirmed by polymerase chain reaction. He had 1.15 G/L peripheral neutrophils, 1.1 G/L peripheral lymphocytes, and mild hypogammaglobulinemia at 6 g/L. Rifampycine and isoniazide treatment was administered for 6 months combined with pyrazinamide during the first 2 months. The clinical evolution was satisfactory with regression of the clinical signs and pulmonary lesions under treatment. However, during follow‐up, the patient developed a *Pseudomonas aeruginosa* infection treated successfully with antibiotics.

**FIGURE 1:**
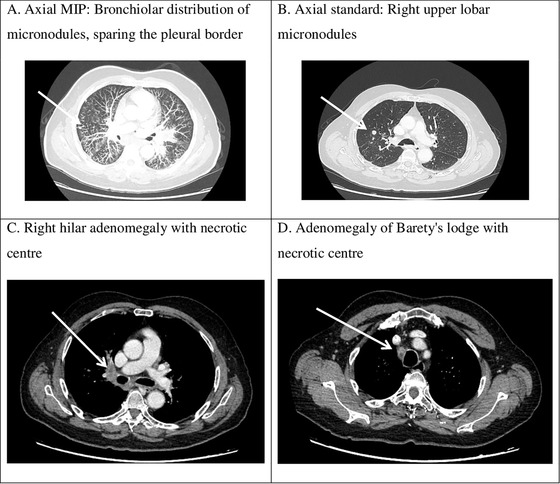
Chest CT scan sections revealing active pulmonary tuberculosis

This case illustrates the occurrence of pulmonary TB in a patient originating from a high endemic region and treated with ibrutinib and rituximab for a MZL. Patients with lymphoid malignancies are particularly at risk of developing infections due to the immune dysfunction inherent to their disease and/or the immunosuppressive effect of their treatments [3]. Pulmonary infections are among the most common [3]. The use of BTKi modifies the frequency and epidemiology of infections [4, 5, 6]. Clinical studies on BTKi reported an incidence of high grade (≥3) severe infections of up to 29%, with higher risks of fungal and bacterial infections in the upper respiratory tract [4, 5, 6]. Indeed, ibrutinib leads to prolonged inhibition of the enzymatic activity of BTK, which is an essential molecule of the signaling pathways of the B‐cell antigen receptor [1]. Ibrutinib also affects the function of T and NK lymphocytes and macrophage phagocytosis [7, 8]. Phagocytosis is crucial for the control of intracellular bacteria, such as *M. tuberculosis*. However, the occurrence of pulmonary TB during ibrutinib treatment has been scarcely reported [9, 10]. Peters et al reported a case of *M. tuberculosis* positive cultures after 2 months of treatment with ibrutinib in a patient with chronic lymphocytic leukemia who had an epidural abscess [9]. Wang et al also reported a patient who developed miliary tuberculosis after initiating ibrutinib [10]. In a recent analysis of four randomized studies of ibrutinib, pneumonia was the only grade 3/4 infection that occurred in more than 3% of patients in either group and mycobacterial infections were also more frequent with ibrutinib when adjusting for exposure [6]. Vigibase, the World Health Organization pharmacovigilance database, identifies 30 individual case safety reports of "mycobacterial infectious disorders" after ibrutinib administration, including 21 tuberculosis cases, mainly with pleuro‐pulmonary localization. The analysis of these cases showed them to involve 13 men and four women (four unknown), ranging from 54 to 91 years of age, and from 1.5 to 7 months since the initiation of treatment. The evolution was fatal in two cases. An association with rituximab is mentioned in two of these cases. The contribution of rituximab, in association with ibrutinib, in the occurrence of TB is questionable, as this agent is generally not considered to be a risk factor for TB [11].

Although one‐third of the world's population is infected with *M. tuberculosis*, 90% develop an effective immune response that is able to control the infection, the pathogen being maintained at the site of primary infection as an asymptomatic latent TB infection (LTBI) [12]. A small proportion (5‐10%) develops active TB. Overall, individuals at high risk of active TB are the contacts of positive cases and those coming from an endemic area [12, 13]. Immune deficiencies induced by HIV infection or immunosuppressive therapy also increase the risk of TB [13]. In particular, anti‐TNF‐α agents, which reduce the production of TNF‐α by macrophages, where mycobacteria survive during LTBI, increase the risk of TB reactivation, mostly resulting from the reactivation of a latent infection [13]. Numerous analyses have consistently shown that patients with LTBI who receive anti‐TNF‐α therapy have an approximately four‐fold increase in the risk of developing active TB over that of healthy individuals [14]. Screening for LTBI should be performed before starting therapy using a dual strategy that includes the traditional tuberculin skin test reaction and an ELISA‐ or ELISPOT‐based interferon‐γ release assay. In this context, anti‐TB therapy should be offered to patients diagnosed with LTBI to reduce the risk of progression to active TB [14, 15].

Thus far, there are no recommendations for patients at risk of TB for whom treatment with ibrutinib is initiated. The present case raises the question of the putative impact of ibrutinib treatment on the onset or reactivation of TB in subjects at risk. Therefore, we recommend that the detection of LTBI should be mandatory in at‐risk patients before initiation of BTKi.

## AUTHOR CONTRIBUTION

Traoré Solo, Roumila Mehdi, and Besson Caroline wrote the paper. Besson Caroline coordinated the research. Pirayeh Eftekhari analyzed the pharmacovigilance data. Hassan Farhat and Fatiha Merabet followed the patient and reported the data. Guira Oumar, Azarian Reza, and Rousselot Philippe interpreted the data and reviewed the letter.

## CONFLICT OF INTEREST

The authors declared no conflict of interest.
